# Osthole Stimulates Osteoblast Differentiation and Bone Formation by Activation of β-Catenin–BMP Signaling

**DOI:** 10.1002/jbmr.21

**Published:** 2010-01-04

**Authors:** De-Zhi Tang, Wei Hou, Quan Zhou, Minjie Zhang, Jonathan Holz, Tzong-Jen Sheu, Tian-Fang Li, Shao-Dan Cheng, Qi Shi, Stephen E Harris, Di Chen, Yong-Jun Wang

**Affiliations:** 1 Spine Research Institute, Shanghai University of Traditional Chinese MedicineShanghai, People's Republic of China; 2 Department of Orthopaedics, Center for Musculoskeletal Research, University of RochesterRochester, NY, USA; 3 Department of Orthopaedics & Traumatology, Longhua Hospital, Shanghai University of Traditional Chinese MedicineShanghai, People's Republic of China; 4 Department of Periodontics, University of Texas Health Science Center at San AntonioSan Antonio, TX, USA

**Keywords:** osthole, β-catenin, BMP-2, osteoblast, osteoporosis, ovariectomy

## Abstract

Osteoporosis is defined as reduced bone mineral density with a high risk of fragile fracture. Current available treatment regimens include antiresorptive drugs such as estrogen receptor analogues and bisphosphates and anabolic agents such as parathyroid hormone (PTH). However, neither option is completely satisfactory because of adverse effects. It is thus highly desirable to identify novel anabolic agents to improve future osteoporosis treatment. Osthole, a coumarin-like derivative extracted from Chinese herbs, has been shown to stimulate osteoblast proliferation and differentiation, but its effect on bone formation in vivo and underlying mechanism remain unknown. In this study, we found that local injection of Osthole significantly increased new bone formation on the surface of mouse calvaria. Ovariectomy caused evident bone loss in rats, whereas Osthole largely prevented such loss, as shown by improved bone microarchitecture, histomorphometric parameters, and biomechanical properties. In vitro studies demonstrated that Osthole activated Wnt/β-catenin signaling, increased *Bmp2* expression, and stimulated osteoblast differentiation. Targeted deletion of the *β-catenin* and *Bmp2* genes abolished the stimulatory effect of Osthole on osteoblast differentiation. Since deletion of the *Bmp2* gene did not affect Osthole-induced *β-catenin* expression and the deletion of the *β-catenin* gene inhibited Osthole-regulated *Bmp2* expression in osteoblasts, we propose that Osthole acts through β-catenin–BMP signaling to promote osteoblast differentiation. Our findings demonstrate that Osthole could be a potential anabolic agent to stimulate bone formation and prevent estrogen deficiency–induced bone loss. © 2010 American Society for Bone and Mineral Research.

## Introduction

Increased longevity and a changing lifestyle have significantly increased the prevalence of osteoporosis, a skeletal disorder characterized by reduced bone mineral density (BMD) and deteriorating bone microarchitecture. Osteoporosis affects over 10 million Americans. Over 1.5 million osteoporotic fractures occur every year and are a major cause of morbidity and mortality with an annual cost of nearly $20 billion in the United States.(1–3) About 40% of women and 13% of men age 50 years or older will experience at least one fracture during their remaining lifetime. The risk factors associated with osteoporotic fracture include age, prior fracture, smoking, and systemic corticosteroid use, and fracture incidence increases with age.(4,5) Aging-associated bone loss can be divided into two phases: a rapid phase starting at menopause and a slow phase occurring about 8 years after menopause and persisting until death. The rapid phase is caused by estrogen insufficiency and mainly affects trabecular bone. In contrast, the slow phase is a process believed to be the result of a more complex, less well understood etiology causing loss of both trabecular and cortical bone and affects both men and women.(6)

Current treatment regimens for osteoporosis fall into two categories: antiresorptive drugs that inhibit osteoclast function such as estrogen and estrogen-receptor analogues and bisphosphates and anabolic drugs that induce osteoblastic bone formation. While estrogen replacement has produced positive results with respect to improved BMD and reduced fracture incidence in early menopause, its prolonged use is restricted because of potential complications such as breast cancer, uterine bleeding, and cardiovascular events. One concern related to the usage of bisphosphates is the complications of osteonecrosis of the jaw (ONJ). The incidence of ONJ disease seems relatively low in patients receiving oral bisphosphates for osteoporosis or Paget's disease and considerably higher in patients with malignancy receiving high doses of intravenous bisphosphates.(7,8) Despite an excellent safety profile for parathyroid hormone (PTH), concerns do arise from its persistence after discontinuation without sequential use of antiresorptive drugs.(9,10) In addition, PTH is not recommended for patients with a risk of osteosarcoma. The belief that combined use of both types of drugs may have a synergistic effect on BMD is not fully supported by some observational studies.(11,12) These potential limitations of existing drugs are sufficient to warrant a development of novel therapies.

Because of the strong association of osteoporosis with heritability, a large number of studies have been performed with either candidate-gene or genome-wide screening strategies. A highly resourced study using a combined linkage and association analysis has linked the *Bmp2* gene to a combined phenotype of low BMD and high fracture risk.(13) Polymorphisms in the *Bmp4* gene are associated with senile osteoporosis.(14) In fact, several small-molecular-weight compounds have been identified by their stimulatory effect on *Bmp2* gene expression. It has been reported that statins stimulate bone formation by upregulation of bone morphogenetic protein 2 (BMP-2) activity.(15) A similar effect is observed with proteosome inhibitors and microtubule inhibitors that induce *Bmp2* expression through inhibition of Glioma-associated oncogene2 (Gli2) and (Gli3) degradation.(16–18) BMP-2 acts on bone cells by binding to their cell surface receptors and subsequently phosphorylating Smads1/5/8. Phosphorylated Smads1/5/8 forms a complex with Smad4 and translocates to the nucleus, whereby it regulates the transcription of bone-specific genes.(19–23) Emerging evidence suggests that BMP-2 has an ameliorative effect on osteoporosis.(24) Therefore, it is desirable to identify compounds capable of inducing endogenous BMP-2 production in bone cells.

To explore the alternative anabolic drugs for the treatment of osteoporosis, this study aimed to investigate the in vivo effects of Osthole, a derivative of Chinese herb medicine, on bone formation using the calvarial local injection mouse model and the ovariectomy rat model. We found that Osthole stimulates new bone formation after its local injection over the surface of calvaria. Osthole is also capable of significantly reversing ovariectomy-induced bone loss in rats. Concerning fragile fracture, we examined the effect of Osthole on the biomechanical properties of the femoral diaphysis in ovariectomized rats and found that it significantly enhanced bone mechanical strength. In vitro studies demonstrated that Osthole-induced osteoblast differentiation through activation of Wnt/β-catenin–BMP signaling in osteoblasts.

## Materials and Methods

### In vivo periosteal bone-formation assay

All surgical protocols related to the use of mice were approved by the University of Rochester Institutional Review Board. Four-week-old ICR Swiss mice were injected subcutaneously over the calvarial surface with or without the treatment of Osthole twice a day for 5 consecutive days at the doses of 1 and 5 mg/kg per day (3 mice per group). Microtubule inhibitor TN-16 (CalBiochem, San Diego, CA, USA) was used as a positive control (5 mg/kg per day, by subcutaneous injection, twice a day for 2 days; 3 mice per group).(17) All mice were euthanized 3 weeks after treatment, and calvariae were dissected, fixed in 10% phosphate-buffered formalin for 2 days, decalcified in 10% EDTA for 2 weeks, and embedded in paraffin. Histologic sections were cut and stained with hematoxylin and eosine (H&E) orange G. New bone area over the calvarial surface was quantified by histomorphometry using the OsteoMeasure System (OsteoMetrics, Inc., Atlanta, GA, USA). To measure mineral appositional rate (MAR) and bone-formation rate (BFR), double calcein labeling was performed at days 7 and 14 by intraperitoneal injection (20 mg/kg), and mice were euthanized 7 days after the second labeling. The labeling was examined in plastic sections. The dissected calvarial samples were fixed in 75% ethanol and embedded in methyl methacrylate. Unstained transverse sections (3 µm thick) were examined with a fluorescent microscope. MAR and BFR were measured using the OsteoMeasure System (OsteoMetrics, Inc.).(25,26)

### Rat ovariectomy surgery

All surgical protocols related to the rat ovariectomy experiment were approved by the Ethical Committee of Shanghai Laboratory Animal Center. Thirty 6-month-old female Sprague-Dawley rats were purchased from the SLAC Laboratory Animal Co., Ltd. (Shanghai, China). After anesthesia with intraperitoneal nembutal injection (30 mg/kg), the rats were randomized by body weight into three groups for the surgery (*n* = 10/group): group 1: sham surgery followed by PBS vehicle treatment (sham + VEH); group 2: ovariectomy followed by vehicle treatment (OVX + VEH); and group 3: ovariectomy followed by Osthole treatment (OVX + OST). The treatment was started 1 month after surgery and continued for 8 weeks. Vehicle or Osthole (100 mg/kg per day) was administered orally once a day for 8 weeks. Before rats were euthanized at the end of the experiments, the total bone mineral density (BMD, g/m^2^) was measured using dual-energy X-ray absorptiometry (Hologic, Inc., Waltham, MA, USA). The fourth lumbar vertebrae (L_4_) then were dissected for histomorphometric and micro-computed tomographic (µCT) analysis, and the left femoral shafts were used for biomechanical testing.

### µCT analysis

The fourth lumbar vertebrae (L_4_) were scanned at 18-µm voxel size using the µCT scanner (µCT80, Scanco Medical AG, Bassersdorf, Switzerland). The trabecular bone under the growth plate was segmented using a contouring tool, and the contours were morphed automatically to segment the trabecular bone on all slices. The 3D images were constructed and analyzed with the evaluation software of the µCT system.

### Histomorphometric analysis

The fourth lumbar vertebrae (L_4_) were fixed in 70% ethanol and embedded in methyl methacrylate. Midsagittal sections (50 µm) were cut with a LEICA SM 2500E Microtome (LEICA, Heidelberger, Germany). Histomorphometric analysis of the trinitrophenol poinsettia–stained sections was performed with an image autoanalysis system (Olympus BX50, Olympus, Tokyo, Japan). The trabecular bone was measured in the area of the secondary spongiosa 0.5 mm apart from the growth plate (2-mm area). All values were represented as the mean of three measurements in three nonconsecutive sections. Histomorphometric parameters were calculated and expressed according to the recommendations made by the American Society for Bone and Mineral Research (ASBMR) nomenclature committee.(27) The image analysis system automatically determined the measuring areas, and the structural parameters were presented as trabecular bone volume (BV/TV, %), trabecular thickness (Tb.Th, µm), trabecular number (Tb.N, 1/mm), and trabecular separation (Tb.Sp, µm).

### Biomechanical testing

After careful removal of soft tissue, the femoral samples were incubated in PBS for 3 hours for thorough hydration. To determine the changes in mechanical properties, the femoral shafts were tested to failure via three-point bending using an Instron Dynamite 8841 servohydraulic material-testing device equipped with a 1-kN load cell, and the data collection was performed with Bluehill software (Instron, Norwood, MA, USA). The anterior surface of the femoral shaft was placed on the two support beams separated by an 8-mm span. The femurs were bent to failure along the mediolateral axis at a rate of 0.10 mm/s. Force and deformation data were analyzed and represented as the bending stiffness (N/mm).(28)

### Primary cell isolation and culture

Calvariae from neonatal mice were removed, subjected to a series of collagenase A (Roche, Indianapolis, IN, USA) digestions at 37°C with shaking, pooled, and then plated on 25 cm^2^ flasks in α modified essential medium (α-MEM, Invitrogen, Carlsbad, CA, USA). Cells were maintained in α-MEM plus 10% fetal bovine serum (FBS) and 1% penicillin-streptomycin (Invitrogen) and were cultured with 5 mM of β-glycerophosphate, 100 mM of l-ascorbic acid, and 0.1 µM of dexamethasone (Sigma, St. Louis, MO, USA) in basic medium for osteoblast differentiation. Cell culture medium was changed every 3 days.

### Cell viability assay

Primary calvarial osteoblasts were seeded in 96-well plates at a density of 0.5 × 10^4^ cells per well. After 2 days of culture, cells were treated with Osthole at concentrations of 0, 1, 5, 10, 50, 100, 500, and 1000 µM for 48 hours (6 wells per group). Cell culture medium then was removed, and 100 µL of CellTiter Blue and medium (Promega, Madison, WI, USA) were added to each well of the plate. After incubated for 4 hours, the fluorescence was read at excitation/emission wavelengths of 560/590 nm in a FLUO-STAR plate reader (Promega).

### Real-time quantitative polymerase chain reaction (qPCR)

Primary calvarial osteoblasts were seeded in 6-well plates at a density of 5 × 10^6^ cells per well. After 2 days of culture, cells were treated with Osthole at concentrations of 10, 50, and 100 µM or BMP-2 (100 ng/mL) (R&D Systems, Minneapolis, MN, USA) for 48 hours (6 wells per group). Total RNA from each well was isolated separately using RNeasy Mini Kit (Qiagen, Valencia, CA, USA). Then 1 µg of RNA from each well was reverse transcribed separately into cDNA using the iScript cDNA Synthesis Kit (Bio-Rad, Hercules, CA, USA). The cDNA then was amplified by PCR (95°C for 15 minutes followed by 45 cycles, 95°C for 20 seconds, 58°C for 20 seconds, and 72°C for 30 seconds) with Absolute QPCR SYBR Green Master Mix (Thermo Scientific, Waltham, MA, USA) in a total volume of 20 µL of buffered solution containing 1 µL of the diluted (1:5) reverse-transcription product in the presence of 10 pM of sense and antisense primers specific for the genes listed in Table 1.

**Table 1:** Mouse Primers for Real-Time qPCR Assays

Genes	Forward primer	Reverse primer
*β-actin*	TGTTACCAACTGGGACGACGACA	CTGGGTCATCTTTTCACGGT
*ALP*	TGACCTTCTCTCCTCCATCC	CTTCCTGGGAGTCTCATCCT
*Bmp2*	ACTTTTCTCGTTTGTGGAGC	GAACCCAGGTGTCTCCAAGA
*Bmp4*	GAGGAGGAGGAAGAGCAGAG	TGGGATGTTCTCCAGATGTT
*Bmp6*	CTCAGAAGAAGGTTGGCTGG	ACCTCGCTCACCTTGAAGAA
*Wnt1*	ACAGCGTTCATCTTCGCAATCACC	AAATCGATGTTGTCACTGCAGCCC
*Wnt3a*	GGCTCCTCTCGGATACCTCT	GGGCATGATCTCCACGTAGT
*Wnt4*	CTCAAAGGCCTGATCCAGAG	GTCCCTTGTGTCACCACCTT
*Col1a1*	CCTGGTAAAGATGGGCC	CACCAGGTTCACCTTTCGCACC
*Runx2*	TCCTGTAGATCCGAGCACCA	CTGCTGCTGTTGTTGCTGTT
*BSP*	AGGACTGCCGAAAGGAAGGTTA	AGTAGCGTGGCCGGTACTTAAA
*OC*	CTTGAAGACCGCCTACAAAC	GCTGCTGTGACATCCATAC

### ALP activity/staining and nodule-formation assay

After treatment with either BMP-2 or Osthole for 48 hours (4 wells per group), primary calvarial osteoblasts were lysed with passive lysis buffer (Promega). Protein concentrations were determined by Coomassie Protein Assay Kit (Bio-Rad) and alkaline phosphatase (ALP) activities were analyzed with solutions containing 0.5 mg/mL of *p*-nitrophenyl-phosphate (Sigma) in AMP buffer (0.5 M 2-methyl-1,2-aminopropanol and 2 mM magnesium chloride, pH 10.3) at 37 °C for 15 minutes. The reaction was stopped by a solution containing 0.3 M sodium phosphate (pH 12.3), and ALP activity data were normalized by the protein concentration. For the ALP staining assay, after treatment with either BMP-2 or Osthole for 48 hours, primary calvarial osteoblasts were fixed in 10% neutral buffered formalin for 15 minutes, washed, and then incubated with ALP staining buffer NBT-BCIP (Bio-Rad) at 37 °C for 30 minutes (*n* = 3).

For the nodule-formation assay, primary calvarial osteoblasts were seeded in 6-well plates at a density of 5 × 10^6^ cells per well and cultured for 2 days in α-MEM supplemented with 100 µg/mL ascorbic acid and 5 mM β-glycerophosphate. Cells then were treated with Osthole at concentrations of 10, 50, and 100 µM or BMP-2 (100 ng/mL) (3 wells per group). Cell culture medium was changed every 3 days, and fresh medium and reagents were added. After 14 days of incubation, cells were stained with 2% alizarin red to analyze formation of mineralized bone nodules.

### Western blot analysis

To examine the effect of Osthole on BMP-2 expression and on BMP signaling, primary calvarial osteoblasts were seeded in 6-well plates at a density of 5 × 10^6^ cells per well. After 2 days of culture, cells were treated with Osthole at concentrations of 10, 50, and 100 µM for 48 hours. Cell lysates were extracted with E-PER protein extraction reagents (Thermo Scientific, Waltham, MA, USA) according to the manufacturer's protocol. Proteins were transblotted onto a PVDF membrane (Bio-Rad), and the membrane was blocked with 5% milk in phosphate buffered saline containing, 0.1% Tween-20 (PBST) for 1 hour at room temperature. After incubation with the primary antibody overnight at 4 °C and the horseradish peroxidase (HRP)–conjugated secondary antibodies (Thermo Scientific) for 1 hours at room temperature, protein expression was detected using a SuperSignal West Femto Maximum Sensitivity Substrate Kit (Thermo Scientific). The polyclonal rabbit BMP-2 antibody was obtained from Abcam (Cambridge, MA, USA), and the polyclonal rabbit anti-β-catenin antibody was obtained from Cell Signaling Technologies (Beverly, MA, USA). After the immunocomplex was removed by stripping buffer (Chemicon, Inc., Temecula, CA, USA), the same membrane was reblotted with mouse anti-β-actin antibody (Sigma) for the loading control. To examine the effect of Osthole on Smad1/5/8 phosphorylation, mouse primary osteoblasts were treated with either Osthole (100 µM) or BMP-2 (100 ng/mL) in the absence or presence of noggin (300 ng/mL, R&D Systems) for 2 hours. After cell lysis, Western blotting was performed using rabbit anti-phospho-Smad1/5/8 polyclonal antibody (Cell Signaling Technologies).

### Immunofluorescence labeling and confocal microscopy

Primary mouse osteoblasts were seeded at a density of 1 × 10^3^ cells per well in 4-well chamber slides (Nalgene, Rochester, NY, USA) for 24 hours and then treated with Osthole (100 µM) or BMP-2 (100 ng/mL) for 2 hours (*n* = 3). After washes with PBS, cells were fixed with acetone/methanol (1:1) at 4 °C for 30 minutes. Nonspecific binding was blocked by incubation with PBS containing 10% normal goat serum at room temperature for 1 hour. After excess serum was removed, rabbit antibody against phospho-Smad1/5/8 (1:50 dilution) in PBS containing 10% goat serum and 0.1% saponin (Research Organics, Inc., Cleveland, OH, USA) was applied to slides and incubated overnight at 4 °C. After thorough washes with PBS, the slides were incubated in the dark for 1 hour with fluorescein isothiocyanate (FITC)–conjugated goat anti-rabbit secondary antibody (1:100 dilution; Jackson ImmunoResearch Laboratories, West Grove, PA, USA) in PBS containing 0.1% saponin and 10% goat serum. The slides then were rinsed with tap water for 30 minutes and mounted with VECTASHIELD medium (Vector Laboratories, Burlingame, CA, USA). Immunofluorescence was detected with a Zeiss microscope using appropriate filters.

### Plasmid transfection and luciferase assay

Primary mouse osteoblasts were seeded at a density of 1 × 10^4^ cells per well in 96-well plates and cultured. The BMP signaling reporter construct 12 × SBE-OC-Luc(29) was transfected into primary osteoblasts using FuGENE HD Transfection Reagent (Invitrogen. An SV40-*Renilla* luciferase construct was cotransfected with the preceding firefly reporter construct as an internal control to normalize transfection efficiency. Cells were incubated with Osthole at concentrations of 0, 10, 50, and 100 µM or BMP-2 (100 ng/mL) for 48 hours (6 wells per group). Cell lysates then were extracted, and luciferase activity was measured using a Promega luciferase assay kit (Promega).

### In vitro deletion of the *β-catenin* and *Bmp2* genes

The *β-catenin*^*fx/fx*^ mice were genotyped and identified by PCR using the following primers: upper primer 5'- AAGGTAGAGTGATGAAAGTTGTT-3' and lower primer 5'- CACCATGTCCTC TGTCTATTC-3' (324-base-pair PCR product). The *Bmp2*^*fx/fx*^ mice were genotyped and identified by PCR using the following primers: wild-type allele: upper primer 5'-agggtttcaggtcagtttccg-3' and lower primer 5'-TCCGAAGGTAAGTGTGCTTGG-3' (200-base-pair PCR product); and *Bmp2*-floxed allele: upper primer 5'-AGGGTTTCAGGTCAGTTTCCG-3' and lower primer 5'-GATGATGAGGTTCTTGGCGG-3' (400-base-pair PCR product). Primary calvarial osteoblasts isolated from the *β-catenin*^*fx/fx*^ and *Bmp2*^*fx/fx*^ mice were seeded in 6-well culture plates at a density of 5 × 10^6^ cells per well and cultured for 2 days (*n* = 3). Adenovirus expressing Cre recombinase (Ad-Cre, 4 × 10^8^ pfu/mL; Baylor College of Medicine, Houston, TX, USA) was added to the cultured osteoblasts for 3 days. Ad-GFP was used as a control and to monitor infection efficiency. After recovery for 2 days, cells were treated with or without Osthole (100 µM) for 48 hours. qPCR and Western blotting were performed to confirm the infection efficiency of Ad-Cre. qPCR was performed to examine changes in mRNA expression of *type I collagen*, *Runx2, OC*, *ALP*, and *OPN*.

### Statistical analysis

Data were expressed as mean ± SEM. Statistical comparisons with results of multiple groups were analyzed using one-way ANOVA followed by Dunnett's test. For experiments involving two groups, unpaired Student's *t* test was performed.

## Results

### Osthole increased new bone formation

**Fig. 1:**
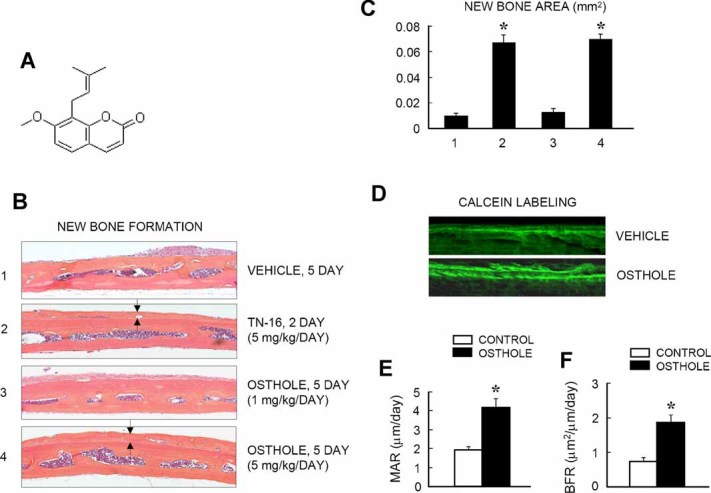
Osthole stimulates local bone formation in mouse calvaria. (*A*) The chemical structure of the coumarin-like structure of Osthole. (*B*, *C*) Osthole was injected subcutaneously over the surfaces of the calvaria (1 and 5 mg/kg/day × 5 days) of 4-week-old ICR Swiss mice. TN-16 was used as a positive control (5 mg/kg/day × 2 days). Mice were euthanized 3 weeks after Osthole injection. H&E orange G staining of calvarial sections showed that Osthole (5 mg/kg/day) significantly induced new bone formation (*black arrows*). (*B*) Histomorphometric analysis showed that Osthole (5 mg/kg/day) significantly increased the total new bone area (group 4). (*C*–*F*) Calcein double labeling was performed in the mice treated with either vehicle or Osthole (5 mg/kg/day × 5 days). Representative pictures taken under ultraviolet (UV) light from undecalcified sections showed that Osthole induced calvarial new bone formation (*D*). Mineral appositional rate (MAR) (*E*) and bone formation rate (BFR) (*F*) were quatified. Significant increases in MAR and BFR in Osthole-treated group were observed. ^*^p < .05, unpaired Student's *t* test (compared with vehicle control).

Osthole is a coumarin derivative and is also known as 7-methoxy-8-isopentenoxycoumarin (Fig. 1*A*). It was extracted and purified from *Cnidium monnieri* and *Angelica pubescens* plants by supercritical CO_2_ method with over 98% purity as determined by HPLC. Subcutaneous injection of Osthole at a dose of 5 mg/kg per day onto mouse calvariae significantly stimulated local bone formation, as shown by histologic analysis of calvarial samples harvested 2 weeks after the last injection and stained with H&E orange G. Histomorphometric analysis revealed that Osthole had a significant effect on bone formation as potent as the positive control, the microtubule inhibitor TN-16.(17) This effect, however, was not seen when Osthole was used at a dose of 1 mg/kg per day (Fig. 1*B, C*). To further confirm whether Osthole stimulates new bone formation on calvariae, Osthole injection (5 mg/kg per day) onto mouse calvariae was followed by calcein double labeling on days 7 and 14. Mice were euthanized 7 days after the second labeling, and histomorphormetric analysis showed a significant difference between control and Osthole-treated groups, demonstrated by an increased mineral appositional rate (MAR) and bone-formation rate (BFR) (Fig. 1*D–F*).

### Osthole prevents bone loss in ovariectomized rats

**Fig. 2:**
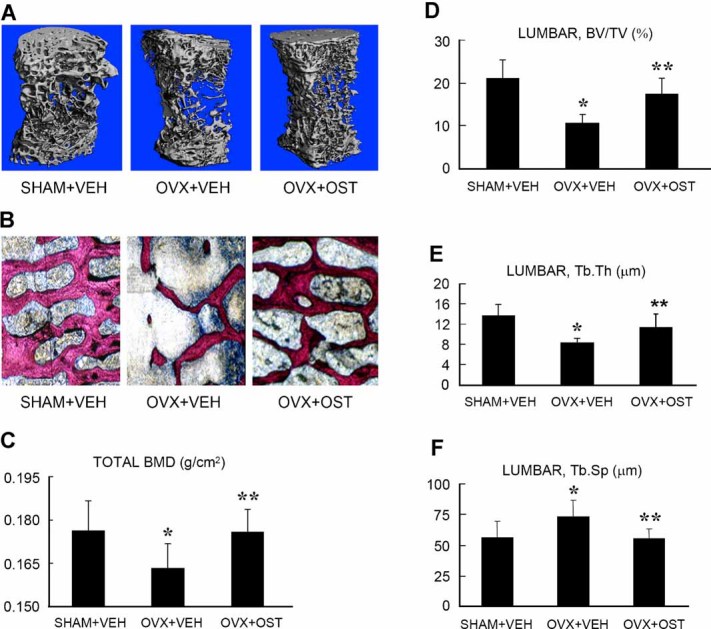
Osthole reverses bone loss induced by ovariectomy in rats. Fourth lumbar vertebrae (L_4_) samples were obtained from the rats of the following groups: (1) sham surgery with vehicle control (sham + VEH), (2) ovariectomy with vehicle control (OVX + VEH), and (3) ovariectomy with Osthole treatment (OVX + OST, 100 mg/kg/day). The Osthole treatment was started 1 month after surgery and lasted for 8 weeks (oral administration, daily treatment). µCT 3D images of L_4_ samples (*A*) and histologic sections of L_4_ samples stained with trinitrophenol poinsettia (*B*) were obtained and analyzed. Bone histomorphometric analysis showed that total BMD (*C*), trabecular bone volume (BV/TV, %) (*D*), trabecular thickness (Tb.Th, µm) (*E*) were significantly increase and trabecular separation (Tb.Sp, µm) (*F*) was significantly reduced in Osthole-treated rats compared with the vehicle-treated control group. ^*^p < .05, unpaired Student's *t* test (OVX + VEH group versus sham + VEH group) and ^**^p < .05, unpaired Student's *t* test (OVX + OST group versus OVX + VEH group).

We then further analyzed the systemic effect of Osthole on ovariectomy-induced bone loss. µCT 3D image analysis of the fourth lumbar vertebrae (L_4_) demonstrated an apparent bone loss in the ovariectomized rats compared with those that underwent sham surgery. Intraperitoneal injection of Osthole for 8 weeks significantly reversed bone loss in the ovariectomized rats (Fig. 2*A*). Histologic examination of the L_4_ samples stained with trinitrophenol poinsettia demonstrated a partial recovery of the trabecular structure in ovariectomized rats treated with Osthole (Fig. 2*B*). Histomorphometric analysis showed that treatment with Osthole significantly increased total BMD, trabecular bone volume, and trabecular thickness and decreased trabecular separation (Fig. 2*C–F*). In addition, osteoclast numbers and osteoclast surface were decreased significantly in rats treated with Osthole (data not shown), suggesting that reduced osteoclast formation also may contribute to the increased bone mass phenotype in Osthole-treated animals.

### Osthole improves biomechanical properties of bone

To further determine if the treatment with Osthole improves biomechanical properties of bone, a three-point bending test was performed on femoral shaft samples obtained from three different groups. Ovariectomy caused a dramatic reduction in maximal force (the maximal force that causes bone fracture), yield force (the applied force that creates permanent bone damage), and bone stiffness (the flexible ability against bone distortion). Treatment with Osthole for 8 weeks significantly reversed the reduced maximal force, yield force, and stiffness caused by ovariectomy (Table 2), indicating that Osthole improves bone biomechanical properties of ovariectomized rats significantly.

**Table 2:** Osthole Improves the Biomechanical Properties of Mouse Shafts (Three-Point Bending Test)

Group	Maximum force (N)	Yield force (N)	Stiffness (N/mm)
1. Sham + VEH	124.9 ± 12.5	106.1 ± 24.3	155.1 ± 64.7
2. OVX + VEH	99.7 ± 15.1*	77.3 ± 37.4*	87.2 ± 24.4*
3. OVX + OST	120.9 ± 13.7**	102.8 ± 23.9**	119.0 ± 33.6**

Mean ± SE.

* *p* < .05, unpaired Student's *t* test (OVX + VEH versus sham + VEH)

** *p* < .05, unpaired Student's *t* test (OVX + OST versus OVX + VEH).

### Osthole stimulates osteoblast differentiation

**Fig. 3:**
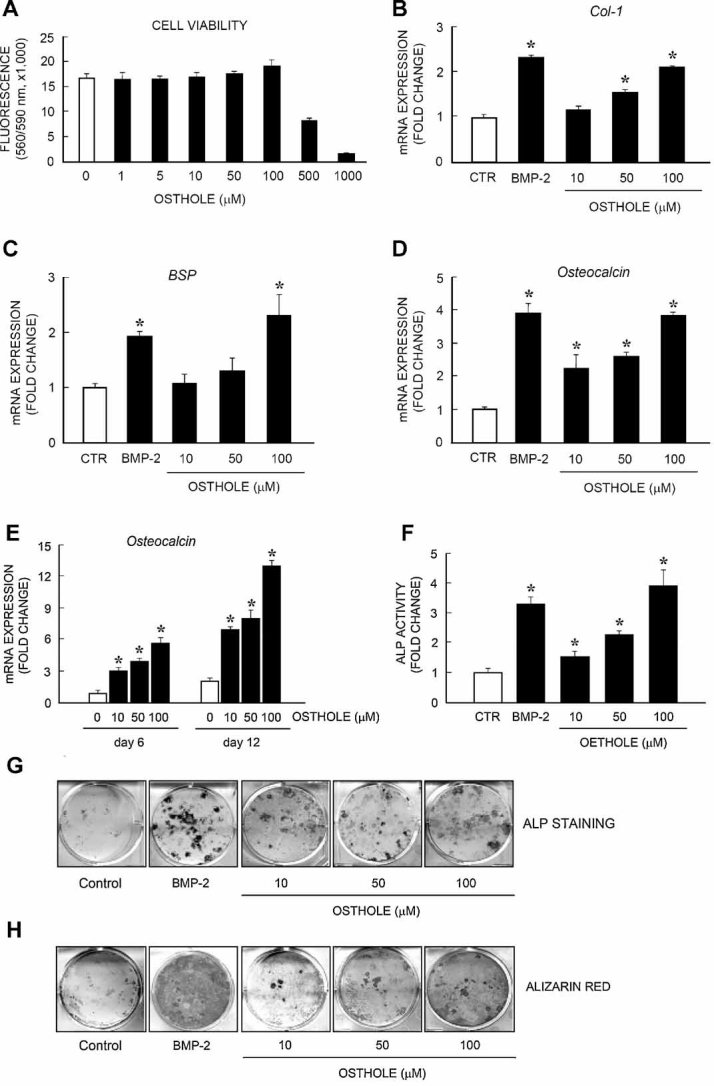
Osthole stimulates osteoblast differentiation. (*A*) Cell viability assay showed that Osthole had no cell toxicity when used at concentrations ranging from 1 to 100 µM. (*B–D*) Osthole (10, 50, and 100 µM) was added to the culture of primary mouse calvarial osteoblasts for 2 days. Real-time qPCR assay was performed to examine changes in expression of osteoblast marker genes including *type I collagen* (*B*), *bone sialoprotein* (*C*), and *osteocalcin* (*D*) (*n* = 6). Osthole significantly increased the expression of osteoblast marker genes in a dose-dependent manner. BMP-2 was used as a positive control. (*E*) The long-term culture of primary mouse osteoblasts further demonstrated that Osthole significantly increased *osteocalcin* expression in day 6 and day 12 cultures in a dose-dependent manner up to 13-fold (*n* = 6). (*F*, *G*) ALP activity (*F*) and ALP staining (*G*) assays were performed. Osthole significantly increased ALP activity in primary osteoblasts (*n* = 3). (*H*) Primary mouse osteoblasts were cultured with or without Osthole (10, 50, and 100 µM) for 14 days. Mineralized bone nodule formation was measured by alizarin red staining. Osthole significantly increased bone nodule formation (*n* = 3). BMP-2 was used as a positive control. ^*^p < .05, one-way ANOVA followed by Dunnett's test (Osthole versus control) and unpaired Student's *t* test (BMP-2 versus control).

Assessment of cell viability did not detect obvious toxicity when Osthole was used at a dose up to 100 µM. However, when the dose reached 500 µM, Osthole started to show toxic effect (Fig. 3*A*). Based on these observations, Osthole was used in all in vitro studies at the dose range of 10 to 100 µM. Osthole dose-dependently promoted osteoblast differentiation, as shown by the upregulation of osteoblast differentiation marker genes such as type I collagen (*col1)*, bone sialoprotein (*BSP*) and osteocalcin (*OC)* (Fig. 3*B–D*) (2 days of culture). The long-term culture (days 6 and 12) showed that Osthole had stronger effect on *OC* expression, with up to a 13-fold increase (Fig. 3*E*). Alkaline phosphatase (ALP) activity is another important indicator for osteoblast differentiation, and we measured ALP activity and performed an ALP staining assay 2 days after treatment with Osthole. We found that Osthole promoted ALP activity in mouse primary osteoblasts in a dose-dependent manner (Fig. 3*F, G*). To determine the effect of Osthole on osteoblast terminal differentiation, we carried out long-term cultures (14 days) using primary mouse osteoblasts and performed alizarin red staining. We found that addition of Osthole to the cell culture significantly enhanced bone nodule formation in primary osteoblasts (Fig. 3*H*).

### Osthole activates BMP signaling

**Fig. 4:**
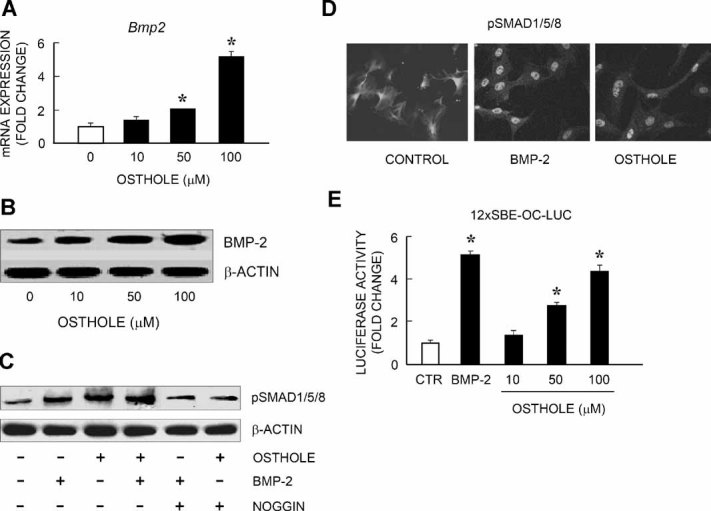
Osthole activates BMP-2 signaling. (*A*, *B*) Primary mouse osteoblasts were cultured with or without Osthole (10, 50, and 100 µM) for 2 days. Expression levels of *Bmp2* mRNA (*A*) and protein (*B*) were measured by real-time PCR and Western blotting. Osthole significantly increased *Bmp2* mRNA and protein expression. (*C*) The effect of Osthole on Smad1/5/8 phosphorylation was examined using an anti-phospho-Smad1/5/8 antibody. Osthole (50 µM) significantly increased Smad1/5/8 phosphorylation. Addition of noggin completely inhibited Osthole-induced Smad1/5/8 phosphorylation. As a positive control, BMP-2 induced Smad1/5/8 phosphorylation. However, Osthole cannot further enhance BMP-2-induced Smad1/5/8 phosphorylation. (*D*) Osthole significantly increased phospho-Smad1/5/8 nuclear translocation in primary osteoblasts, demonstrated by immunofluorescence labeling using the anti-phospho-Smad1/5/8 antibody. (*E*) In addition, Osthole (50 and 100 µM) significantly increased luciferase activity of BMP signaling reporter 12×SBE-OC-Luc (*n* = 6). ^*^p < .05, one-way ANOVA followed by Dunnett's test (Osthole versus control) and unpaired Student's *t* test (BMP-2 versus control).

BMPs have been shown to play an important role in osteoblast differentiation. To determine the mechanism of Osthole effect on osteoblast differentiation, we examined the effect of Osthole on *Bmp* expression and BMP signaling. We found that Osthole enhanced *Bmp2* mRNA and protein expression in a dose-dependent manner (Fig. 4*A, B*). In contrast, Osthole had no significant effect on the expression of *Bmp4* and *Bmp6*, at least at the mRNA expression level (data not shown). To determine if Osthole activates downstream signaling molecules of BMP, we examined the effect of Osthole on Smad protein phosphorylation. Primary mouse osteoblasts were treated with 100 µM of Osthole. The results demonstrated that Osthole significantly enhanced phosphorylation of Smad1/5/8 in osteoblasts (Fig. 4*C*). BMP-2 was used as a positive control and also showed stimulation of Smad1/5/8 phosphorylation. Addition of noggin, a BMP antagonist, completely inhibited BMP-2- and Osthole-induced Smad1/5/8 phosphorylation (Fig. 4*C*), indicating that Osthole-induced Smad1/5/8 phosphorylation is due to activation of BMP expression. Immunofluorescent labeling showed that Osthole also induced a rapid nuclear translocation of phospho-Smad1/5/8 2 hours after treatment (Fig. 4*D*). To further confirm the role of Osthole on BMP signaling, we also examined the effect of Osthole on the BMP signaling reporter 12 × SBE-OC-Luc. Consistent with the preceding findings, Osthole significantly stimulated the luciferase activity of the BMP signaling reporter in a dose-dependent manner (Fig. 4*E*). Taken together, these findings demonstrate that Osthole may regulate osteoblast differentiation through activation of BMP signaling.

### Osthole activates canonical Wnt/β-catenin signaling

**Fig. 5:**
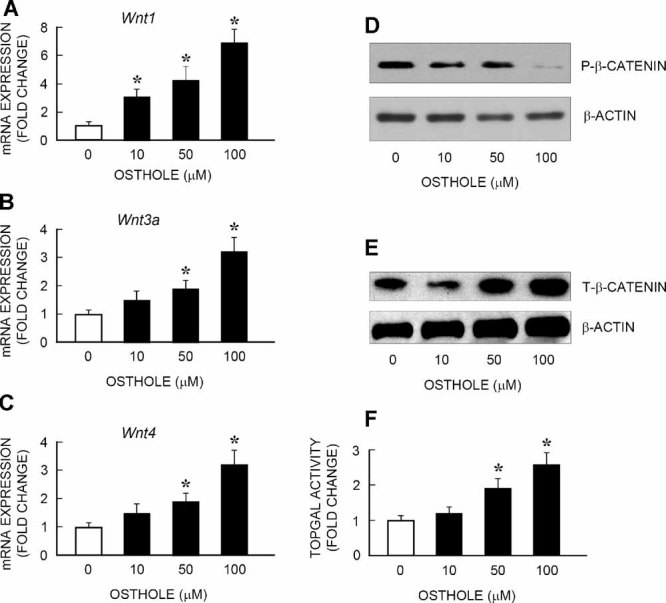
Osthole stimulates canonical Wnt signaling. (*A–D*) Primary mouse osteoblasts were cultured with or without Osthole (10, 50 and 100 µM) for 2 days. Expression levels of *Wnt1*, *Wnt3a*, and *Wnt4* mRNA (*A–C*) and protein levels of phosphorylation and total β-catenin (*D*) were measured by real-time PCR and Western blotting. Osthole significantly increased mRNA expression of *Wnt1*, *Wnt3a*, and *Wnt4* (*A–C*). Osthole also significantly inhibited phospho-β-catenin and increased total β-catenin protein levels (*D*). (*E*) To examine the effect of Osthole on β-catenin signaling, the effect of Osthole on the activity of β-catenin signaling reporter TOPGAL also was examined. Osthole significantly increased luciferase activity of TOPGAL reporter. ^*^p < .05, unpaired Student's *t* test (compared with vehicle control).

Our recent studies demonstrate that *Bmp* expression is activated by canonical Wnt/β-catenin signaling in chondrocytes and in bone marrow stromal cells.(28,30,31) To determine if Osthole affects Wnt/β-catenin signaling, we examined the effects of Osthole on mRNA expression of Wnt ligands, β-catenin protein expression, and β-catenin signaling reporter. We found that Osthole induced *Wnt1, Wnt3a*, and *Wnt4* expression in a dose-dependent manner (Fig. 5*A–C*). As a consequence, Osthole also reduced phosphorylated β-catenin protein levels and enhanced total β-catenin protein levels in primary calvarial osteoblasts (Fig. 5*D, E*). High doses of Osthole (50 and 100 µM) also increased TOPGAL (β-catenin signaling reporter) activity (Fig. 5*F*). As a consequence of activation of β-catenin signaling, the expression of β-catenin downstream target genes, such as *axin2* and *dkk1*, also was increased (data not shown). These findings demonstrate that Osthole also activates canonical Wnt/β-catenin signaling in osteoblasts.

### Osthole-induced osteoblast differentiation is β-catenin- and BMP-2-dependent

**Fig. 6:**
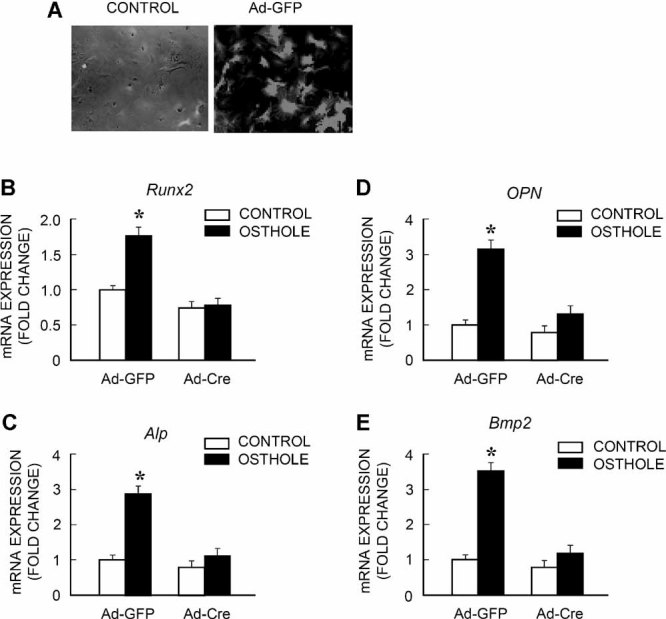
Osthole induces osteoblast differentiation in a β-catenin-dependent manner. Primary mouse osteoblasts were isolated from *β-catenin^fx/fx^* mice, infected with either Ad-GFP or Ad-Cre, and then treated with or without Osthole. Ad-GFP infection showed that primary osteoblasts were infected with adenovirus with high efficiency (>80%) (*A*). The infection of Ad-Cre completely inhibited Osthole-induced *Runx2*, *ALP*, *BSP*, and *Bmp2* expression (*B–E*). ^*^p < .05, unpaired Student's *t* test (compared with Ad-GFP group).

**Fig. 7:**
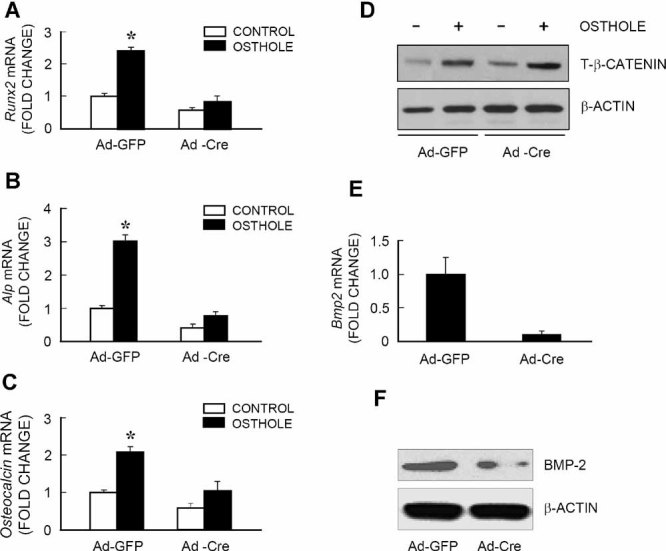
Osthole induces osteoblast differentiation through activation of *Bmp2*. Primary mouse osteoblasts were isolated from *Bmp2^fx/fx^* mice, infected with either Ad-GFP or Ad-Cre and then treated with or without Osthole. Osthole significantly increased *Runx2*, *ALP*, and *OC*, and infection of Ad-Cre significantly inhibited Osthole-induced *Runx2*, *ALP*, and *OC* expression in osteoblasts (*A–C*). However, Ad-Cre infection had no significant effect on β-catenin protein levels (*D*). Infection efficiency of Ad-Cre was confirmed by significant reduction in *β-catenin* mRNA and protein expression (*E*, *F*). ^*^p < .05, unpaired Student's *t* test (compared with Ad-GFP group).

To further determine if the effect of Osthole on osteoblast differentiation depends on its stimulatory effect on β-catenin and BMP-2 signaling, we used the in vitro gene-deletion approach. Primary osteoblasts were isolated from the calvaria of the *β-catenin*^*fx/fx*^ or *Bmp2*^*fx/fx*^ mice and infected with either Ad-GFP or Ad-Cre. The cells then were treated with Osthole for additional 2 days, and the effects of Osthole on the expression of osteoblast marker genes were examined. Ad-GFP infection revealed that high infection efficiency (>80%) was achieved in primary osteoblasts (Fig. 6*A*). Ad-Cre infection in primary osteoblasts isolated from the *β*-*catenin*^*fx/fx*^ mice inhibited Osthole-induced expression of *Runx2*, *ALP*, and *BSP*, as well as *Bmp2* (Fig. 6*B–E*). In contrast, Ad-Cre infection in primary osteoblasts isolated from the *Bmp2*^*fx/fx*^ mice significantly inhibited Osthole-induced expression of *Runx2*, *ALP*, and *OC* (Fig. 7*A–C*) but had no effect on *β-catenin* expression (Fig. 7*D*). Ad-Cre-mediated deletion of the *Bmp2* gene was demonstrated by the reduction of *Bmp2* mRNA and protein expression in these cells (Fig. 7*E, F*). These results suggest that Osthole induces osteoblast differentiation through activation of β-catenin and BMP-2 signaling. Since deletion of the *β-catenin* gene inhibited Osthole-induced *Bmp2* expression but Osthole-regulated *β-catenin* expression was not affected by deletion of the *Bmp2* gene, these findings also suggest that *Bmp2* may act downstream of β-catenin signaling, and Osthole activates osteoblast differentiation through β-catenin–BMP-2 signaling pathway.

## Discussion

Osthole (7-methoxy-8-isopentenoxycoumarin) is a coumarin derivative that is present in many plants such as *Cnidium monnieri* and *Angelica pubescens*. In traditional Chinese medicine, these plants have been used as tonics and aphrodisiacs and for the treatment of bone-related diseases.(32,33) In a rat osteoporosis model induced by ovariectomy, Osthole prevents bone loss through an estrogen-like effect.(34) In addition to Osthole, other coumarin derivatives from *C. monnieri* also have demonstrated an ability to induce bone formation.(35) Osthole has been demonstrated to be able to stimulate proliferation in osteoblast-like UMR106 cells.(36) In vitro study also shows that Osthole has no effect on cell proliferation to human osteoblast-like MG-63 cells or human fetal osteoblasts. Instead, Osthole stimulates osteoblast differentiation.(37) Although the effect of Osthole on osteoblast differentiation has been suggested to be mediated by BMPs, the detailed molecular mechanism has not been clearly defined.

This study has demonstrated the in vivo effect of Osthole by local and systemic administrations of Osthole into mice and rats. We found that Osthole induces new bone formation on the surfaces of mouse calvaria. Histomorphometric analysis shows that Osthole accelerates osteoid formation and mineralization. To more closely mimic the clinical scenario of the treatment of postmenopausal osteoporosis, we started systemic Osthole administration 4 weeks after ovariectomy and continued treatment for 8 weeks. The effects of Osthole on the structural and biomechanical properties of the bone in ovariectomized rats were monitored with µCT, histomorphometric, and three-point bending analyses. While ovariectomy significantly reduced bone mass and strength, systemic Osthole administration largely prevented its deleterious effects. The observation that Osthole improved the biomechanical properties of bone in ovariectomized rats is of particular importance with regard to the treatment of fragile fracture in osteoporotic patients. The safety profile further supports the clinical application of Osthole as a novel anabolic agent for the treatment of osteoporosis.

To determine the mechanism by which Osthole promotes osteoblast differentiation, we examined the effect of Osthole on primary mouse calvarial osteoblasts by monitoring maturation marker genes, ALP activity, and bone nodule formation. Collectively, our in vitro studies indicate that Osthole stimulates osteoblast differentiation and maturation. In these studies, we have demonstrated that Osthole activates both canonical Wnt/β-catenin and BMP-2 signaling in osteoblasts. We found that deletion of either the *β-catenin* or *Bmp2* gene in osteoblasts significantly inhibited Osthole-induced osteoblast differentiation. Since deletion of the *β-catenin* gene completely inhibited Osthole-induced *Bmp2* expression, whereas deletion of the *Bmp2* gene did not affect Osthole-regulated *β-catenin* expression in osteoblasts, these findings suggest that *Bmp2* may serve as a downstream target gene of β-catenin signaling in osteoblasts. Osthole may stimulate osteoblast differentiation through activation of Wnt/β-catenin–BMP-2 signaling pathway.

Targeted deletion of the *Bmp2* gene only partially reverses the effects of Osthole on osteoblast maturation. Several reasons for this may exist. First, Osthole may induce Smad1/5/8 phosphorylation by activating the MAP kinase pathway, as suggested by Kuo and colleagues, and acts downstream of the *Bmp2* gene. Second, other BMPs may play a role in Osthole-induced osteoblast differentiation. Indeed, our qPCR results show that Osthole also upregulates the expression of *Bmp7* mRNA, although it has no obvious effect on the expression of *Bmp4* and *Bmp6*.

In summary, this study clearly demonstrates that Osthole induces new bone formation and prevents bone loss caused by estrogen deficiency, and such effects are mediated by inducing osteoblast differentiation. The end results of this orchestrated activity are improved BMD and biomechanical properties. Osthole may activate β-catenin–BMP-2 signaling pathway to regulate osteoblast differentiation. Our future research will focus on optimizing the pharmacokinetics and pharmacodynamics of Osthole for possible oral administration.
